# A Deep Learning Method for Bearing Cross-Domain Fault Diagnostics Based on the Standard Envelope Spectrum

**DOI:** 10.3390/s24113500

**Published:** 2024-05-29

**Authors:** Lubin Zhai, Xiufeng Wang, Zeyiwen Si, Zedong Wang

**Affiliations:** 1 College of Mechanical Engineering, Xi’an Jiaotong University, Xi’an 710049, China; zlb_5200@stu.xjtu.edu.cn (L.Z.); 2860245534@qm sjhvej@163.com (Z.W.); 2 School of Mathematics, University of Bristol, Bristol BS8 1QU, UK; iy22624@bristol.ac.uk

**Keywords:** rolling bearings, cross-domain fault diagnostics, standardized envelope spectrum, convolutional neural networks

## Abstract

Intelligent fault diagnostics based on deep learning provides a favorable guarantee for the reliable operation of equipment, but a trained deep learning model generally has low prediction accuracy in cross-domain diagnostics. To solve this problem, a deep learning fault diagnosis method based on the reconstructed envelope spectrum is proposed to improve the ability of rolling bearing cross-domain fault diagnostics in this paper. First, based on the envelope spectrum morphology of rolling bearing failures, a standard envelope spectrum is constructed that reveals the unique characteristics of different bearing health states and eliminates the differences between domains due to different bearing speeds and bearing models. Then, a fault diagnosis model was constructed using a convolutional neural network to learn features and complete fault classification. Finally, using two publicly available bearing data sets and one bearing data set obtained by self-experimentation, the proposed method is applied to the data of the fault diagnostics of rolling bearings under different rotational speeds and different bearing types. The experimental results show that, compared with some popular feature extraction methods, the proposed method can achieve high diagnostic accuracy with data at different rotational speeds and different bearing types, and it is an effective method for solving the problem with cross-domain fault diagnostics for rolling bearings.

## 1. Introduction

Rolling bearings are an important part of rotating machinery and equipment, and they are easily damaged under long time operation and bad working conditions. Sudden failure will affect the normal operation of the equipment, resulting in economic losses and even casualties [1]. Therefore, it is of great importance to monitor and diagnose the operating condition of rolling bearings.

Signal processing and intelligent diagnostic methods have been successively applied to rolling bearing fault diagnostics, which has attracted the attention of a large number of scholars due to the fact that intelligent diagnostic methods do not require specialized technicians. Intelligent diagnostic methods usually include two steps: feature extraction and fault classification. Feature extraction is a signal processing method based on the time domain (TD), frequency domain (FD), and time–frequency domain (TFD) [2,3] to extract feature indicators that can characterize the health state. Traditional machine learning methods, such as support vector machines (SVM) [4], principal component analysis (PCA) [5], and artificial neural networks (ANN) [6], have been widely used for fault classification in intelligent diagnostics. However, due to their shallow architectures, they have difficulty learning effective features from raw signals, and the diagnostic performance relies on expert a priori knowledge and signal analysis tools.

Compared with traditional machine learning, deep learning models can automatically discover deep features in the original signals, and have received extensive attention and research in recent years. Deep learning algorithms, such as the convolutional neural networks (CNN) [7], the recurrent neural networks (RNN) [8], th deep autoencoder (DAE) [9] and the deep belief network (DBN) [10], have been successfully applied to bearing fault diagnostics.

CNN is a typical deep learning algorithm with a strong local feature extraction capability and performs well in image recognition and classification tasks. Hoang et al. [11] converted one-dimensional time-domain vibration signals into two-dimensional vibration images and then used CNN for vibration image classification to identify bearing faults. Zhang et al. [12] proposed an improved CNN model using time–frequency images as input for bearing fault diagnosis that is highly adaptable to workload variations. Hasan et al. [13] fused the multi-domain information of raw bearing vibration signals into a two-dimensional composite image, and then fed the composite image into a multi-task learning (MTL)-based CNN model for fault diagnostics, which was able to accurately detect faults in the presence of simultaneous changes in speed and health conditions. Chen et al. [14] used cyclic spectral analysis to construct a frequency domain graph as an input to CNN to reveal the hidden periodic behavior of each fault type in bearing vibration signals, which reduces the difficulties with feature learning in deep diagnostic models. Sobie et al. [15] sequentially performed envelope extraction, simultaneous angular domain averaging, and normalization of the bearing vibration signals before feeding them into a CNN, which allows the fault classification of experimental data with different shaft speeds and bearing geometries.

Although the CNN-based rolling bearing intelligent diagnostic methods have achieved remarkable results, there are still some problems:

(1) Although some feature extraction methods based on the time domain, frequency domain, and time–frequency domain have been applied to intelligent diagnostics, these feature extraction methods do not fully consider the a priori knowledge of the local fault characteristics of the bearings, have poor robustness, and are ineffective in cross-domain diagnostics.

(2) Many existing feature extraction methods do not take into account the differences between source and target domains caused by changes in bearing speeds and bearing models, making it difficult for deep learning models to learn common features between domains when performing cross-domain diagnostics, and degrading the diagnostic performance.

To solve the above problems, a standard envelope spectrum is constructed based on the envelope spectrum morphology of rolling bearing faults. The standard envelope spectrum reveals the signal characteristics of rolling bearings that do not vary with rotational speed and model, but only with changes in health state. That is, the difference between the source and target domains due to different speeds and models is eliminated by taking into account the a priori knowledge of experts. A standard sample library reflecting the health state of rolling bearings is established from the existing bearing data set, and fault diagnostics without target domain samples are realized. A convolutional neural network model is constructed to learn the common features between the source and target domains and perform fault classification by taking the standard envelope spectrogram as input.

The main contributions of this paper can be summarized as follows:

(1) A standard envelope spectrum is constructed that reveals the unique characteristics of different bearing health states.

(2) An intelligent diagnostic method based on vibration signals for the SES-CNN of rolling bearings is proposed, which is effective in the cross-domain diagnostics of bearing data with different rotational speeds and different models.

(3) The proposed method focuses on effective feature representation for the cross-domain fault diagnostics of rolling bearings, establishes a standard sample library reflecting the health state of the rolling bearings, and the diagnostic process does not require target domain samples and models of high complexity.

The rest of the paper is organized as follows: in the next section, the relevant theoretical background is introduced. Section 3 analyzes and presents the proposed framework for rolling bearing fault diagnostics. Section 4 describes the datasets used and provides a comprehensive evaluation and comparison of the methods. Finally, some conclusions are given in Section 5.

## 2. Theoretical Basis

### 2.1. Envelope Spectrum

When the rolling bearing element surface produces local defects, in the rolling body and the inner and outer ring, mutual motion processes will produce periodic impact vibrations, and the frequency of vibration is called failure characteristic frequency. The failure characteristic frequency depends on the shaft speed and bearing type (geometry). Different component failures correspond to different failure characteristic frequencies. The formulas for the outer ring failure characteristic frequency, inner ring failure characteristic frequency, rolling body failure characteristic frequency, and cage failure characteristic frequency calculation are as follows:

(1) $$f_{o}=0.5zf_{r}(1-\frac{d}{D}\cos\alpha )$$

(2) $$f_{i}=0.5zf_{r}(1+\frac{d}{D}\cos\alpha )$$

(3) $$f_{b}=\frac{1}{2}\frac{D}{d}f_{r}\left[1-{\left(\frac{d}{D}\right)}^{2}\cos^{2}\alpha \right]$$

(4) $$f_{c}=0.5f_{r}(1-\frac{d}{D}\cos\alpha )$$

where z is the number of balls, $f_{r}$ is the rotation frequency, d is the ball diameter, D is the raceway pitch diameter, and α is the contact angle.

Envelope spectrum analysis is an effective method for rolling bearing fault diagnostics [16]. Usually, the original signal undergoes Hilbert demodulation to obtain the envelope signal, and then the envelope signal undergoes a Fourier transform to obtain the envelope spectrum.

Let x(t) represent a vibration signal whose analytical signal is expressed by the Hilbert transform as:

(5) $$\tilde{x}(t)=x(t)+jH\{x(t)\}=x(t)+j\frac{1}{\pi }\int_{-\infty }^{+\infty }\frac{x(\tau )}{t-\tau }d\tau$$

where H{⋅} denotes the Hilbert transform and j is the imaginary unit.

The envelope signal is then obtained from the following equation:

(6) $$Env(t)=\left|a(t)\right|=\sqrt{{\left(x(t)\right)}^{2}+{\left(H\{x(t)\}\right)}^{2}}$$

The envelope spectrum is obtained by applying a Fourier transform to the envelope signal, which is given by the following equation:

(7) $$Es(f)=F\left\{\left|a(t)\right|\right\}=\int_{-\infty }^{+\infty }\left|a(t)\right|e^{-j2\pi ft}dt$$

where F{⋅} denotes the fast Fourier transform (FFT).

**Figure 1:**
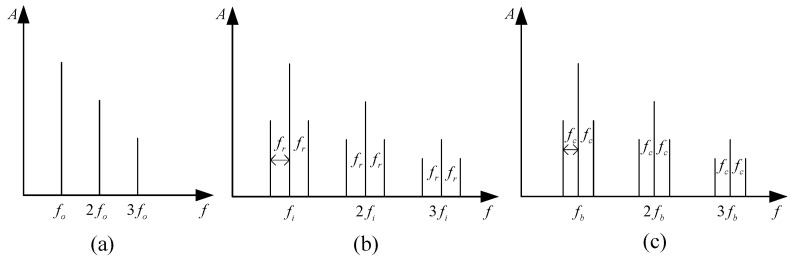
Bearing fault envelope spectrum morphology: (**a**) Outer ring fault; (**b**) Inner ring fault; (**c**) Rolling element fault.

The envelope spectrum can effectively reveal the failure characteristic frequency of rolling bearings, and the expected envelope spectrum patterns of different components of rolling bearings when failures occur are shown in Figure 1 [17]. For the outer ring fault, the main frequency components in the envelope spectrum are the outer ring fault characteristic frequency and harmonics; there is no sideband. For the inner ring fault, the main frequency components in the envelope spectrum are the inner ring fault eigenfrequency and harmonics, and the sideband interval is the rotating frequency sideband. For rolling element faults, the main frequency components in the envelope spectrum are the rolling element fault eigenfrequency and harmonics, and the sideband interval is the sideband of the cage fault eigenfrequency.

### 2.2. Convolutional Neural Networks

A convolutional neural network (CNN) is a deep learning model that performs well at visual recognition tasks, such as image classification and target detection. A typical CNN architecture consists of multiple layers, such as convolutional, pooling, and fully connected layers, which perform different functions and are primarily used to extract features from input data and perform classification [18].

The basic block of the CNN feature extraction part is the convolutional layer. The convolutional layer applies a set of convolutional kernels (also known as learnable filters) to the input data, with each kernel extracting specific features. The efficiency of feature extraction is significantly affected by the filter size, the convolutional step size, and the number of filters used. The convolutional layer captures patterns and structures by sliding these filters over the input data. This operation allows the network to learn a hierarchical representation, starting with simple features, such as edges and corners, and gradually evolving to more complex features. The convolution formula is:

(8) $$y_{l,j}^{conv}=\sum_{i=1}^{k}w_{i,j}^{l}*y_{l-1,i}^{pool}+b_{j}^{l}$$

where $y_{l,j}^{conv}$ represents the convolutional value of the jth channel in convolutional layer l, $y_{l-1,i}^{pool}$ represents the ith channel output in pooling layer l−1, $w_{i,j}^{l}$ represents the kernel of convolutional layer l, $b_{j}^{l}$ represents the bias of the jth channel in the convolutional layer l, and ∗ represents the convolutional operation.

After the convolution operation is completed, whether or not the neurons in the convolution layer are awakened depends on the activation function. Activation functions such as ReLU (Rectified Linear Units) can introduce nonlinearities, promote sparsity, improve gradient propagation, and enable the network to capture complex patterns, which ultimately improves the overall performance and learning ability of convolutional neural networks:

(9) yl,jRelu=f(yl,jconv)=max[0,yl,jconv]

where yl,jRelu represents the jth channel output in the convolutional layer l, and f(⋅) represents the activation function.

In the feature extraction part, several convolutional layers are always followed by a pooling layer, i.e., a sampling layer. The main purpose of the pooling layer is to reduce the dimensionality of the feature maps and to reduce the number of feature maps, thus reducing the computational complexity. Maximum pooling and average pooling are commonly used pooling operations. The maximum pooling function is as follows:

(10) yl,jpool=max(w(s1,s2)∩yl−1,jRelu)

where $w(s_{1},s_{2})$ represents the pooling window, which can slide with a certain step, $s_{1}$ and $s_{2}$ correspond to the dimension of the pooling window, yl−1,jRelu represents the jth channel output in the convolutional layer l−1, and ∩ represents the overlap between the pooling window and the channel output.

The final part of a CNN usually consists of fully connected layers. These layers act as classifiers, taking the high-level representations extracted from the previous layer and mapping them to the target classes. The output layer is usually a softmax layer that generates class probabilities. The formula for the fully connected layer is as follows:

(11) $$y=\sigma ({\left(w_{f}\right)}^{T}s_{m}+b_{f})$$

where $w_{f}$ represents the weight matrix used to connect the two fully connected layers, $b_{f}$ represents the bias, $s_{m}$ represents the input data of the fully connected layer, and σ(⋅) represents the activation function in the fully connected layer.

## 3. The Proposed SES-CNN Model

### 3.1. Construction of Standard Envelope Spectrum (SES)

**Figure 2:**
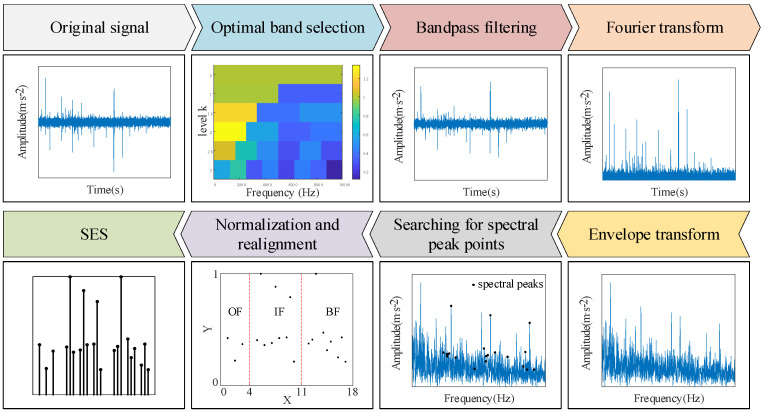
The process of constructing a standard envelope spectrum.

The envelope spectra of rolling bearings are characterized differently when localized defects occur in different elements of the bearing. However, the distribution of spectral lines in the envelope spectrogram changes due to the fact that different types of bearings have different failure characteristic frequencies. Similarly, changes in rotational speed change the distribution of spectral lines in the envelope spectra. Therefore, a standard envelope spectrum is constructed, and the spectral lines of the relevant fault characteristic frequencies in the envelope spectrum are fixed at the specified positions, so that the spectrum not only retains the characteristic differences of different component faults, but also eliminates the differences in the distribution of the characteristic spectral lines caused by changes in rotational speed and bearing models, and has the characteristic that it does not change with changes in bearing rotational speed and models, but only changes due to changes in health. The process of constructing the standard envelope spectrum is shown in Figure 2 with the following steps:

Step 1: To improve the signal-to-noise ratio, a fast kurtogram method is used to select the optimal frequency band of the signal, then a bandpass filter is applied to the optimal frequency band to obtain the filtered signal.

Step 2: A Hilbert transform is applied to the filtered signal to obtain the envelope signal.

Step 3: A Fourier transform is applied to the envelope signal to obtain the envelope spectrum.

Step 4: The fault characteristic frequency of each component of the bearing is calculated using Equations (1)–(4). First, take the outer ring fault characteristic frequency and its harmonic $nf_{o}$, the inner ring fault characteristic frequency and its harmonic $nf_{i}$, and the rolling element fault characteristic frequency and its harmonic $nf_{b}$ as the search center frequency. Next, set the frequency search range according to the form of [F(1−α),F(1+α)], and extract the point with the largest amplitude in the search range of the search center frequency in the envelope spectrum as the spectral peak point. Then, the frequency corresponding to the spectral peak point at the inner ring fault characteristic frequency and its harmonic $nf_{i}$ is taken as the actual inner ring fault characteristic frequency and its harmonic, and these frequencies are taken as the new search center frequency. The frequency search range is set in the form of $\left[F-f_{r}(1+\alpha ),F-f_{r}(1-\alpha )\right]$ and $\left[F+f_{r}(1-\alpha ),F+f_{r}(1+\alpha )\right]$, and the point with the largest amplitude in the search range of the search center frequency in the envelope spectrum is extracted as the spectral peak point. Similarly, the frequencies corresponding to the spectral peaks at the characteristic frequencies and harmonics of the rolling body ring faults and their harmonics $nf_{b}$ are taken as the actual rolling body fault characteristic frequencies and their harmonics. These frequencies are taken as the new search center frequencies, and the frequency search ranges are set in the form of $\left[F-f_{c}(1+\alpha ),F-f_{c}(1-\alpha )\right]$ and $\left[F+f_{c}(1-\alpha ),F+f_{c}(1+\alpha )\right]$, and the point with the largest amplitude in the search center frequency search range of the envelope spectrum is extracted as the spectral peak point. After the above search, a total of 21 spectral peaks $\left(F_{i},a_{i}\right)$ are obtained where 1≤n≤3 and 1≤i≤21, and both n and i are integers. That is, a total of 21 frequency components are considered, including the bearing outer ring fault, the inner ring fault, the rolling element fault characteristic frequency of the first 3 harmonics, as well as the inner ring fault, and the rolling element fault characteristic frequency of the first 3 harmonics around the first-order modulation sidebands. Due to the influence of factors such as bearing slippage, the bearing fault characteristic frequency calculated by kinematics theory and the actual fault frequency often have errors, so this paper considers the error coefficient of α=0.015.

Step 5: Normalize the amplitude of the previously searched peak points $\left(F_{i},a_{i}\right)$ of the spectrum using the following formula:

(12) $$A_{i}=\frac{a_{i}}{a_{i\max}}$$

where 1≤i≤21 and $a_{i\max}$ is the maximum value in $a_{i}$.

Step 6: Establish a new coordinate system where the vertical coordinate range is 0–1 and the horizontal coordinates, from left to right, are the outer ring fault (OF), inner ring fault (IF), rolling element fault (BF), being the three fault characteristics of the region. The normalization of the spectral peak point $\left(F_{i},A_{i}\right)$ is placed in the corresponding fault characteristics of the region, in accordance with the order of $F_{i}$ from small to large, arranged in the corresponding horizontal coordinate position, to generate a new spectral line graph known as the standard envelope spectrum.

Step 7: Save the resulting standard envelope spectrum as a grayscale image in a jpg format with a resolution of 64×64 px.

### 3.2. The Architecture of the Proposed CNN

The CNN model constructed in this paper consists of two convolutional layers, two batch normalization layers, two pooling layers, one spreading layer, and two fully connected layers, where the convolutional layers are used for feature extraction, the pooling layer is used to reduce the spatial dimensionality of the feature maps, the batch normalization layer is used to prevent overfitting and speed up convergence, the spreading layer is used to spread the multidimensional feature maps into one-dimensional vectors, and the fully connected layer is used for classification and output prediction. The size of the convolutional layer filter is (3, 3), the step size is (1, 1), the same filling method is chosen, ReLU is chosen as the activation function, the number of the first convolutional layer filter is 16, and the number of the second convolutional layer filter is 32. Both convolutional layers are followed by a batch normalization layer and a pooling layer where the type of the pooling layer is the maximum pooling layer, the size of the filter is (2, 2), the step size is (2, 2), the size of the filter is (2, 2), and the same padding method is chosen. The output is then flattened by a spreading layer and mapped to the output categories by two fully connected layers, where the number of neurons in the first fully connected layer is 128 and ReLU is chosen as the activation function, and the number of neurons in the second fully connected layer is four and softmax is chosen as the activation function. The model is trained using the ADAM optimizer, the cross-entropy loss is used as the loss function, and the accuracy and loss value are used as metrics to evaluate the training performance of the network model. The details of the layer type and the parameters used are shown in Table 1.

### 3.3. Fault Diagnosis Framework Based on SES and CNN

**Figure 3:**
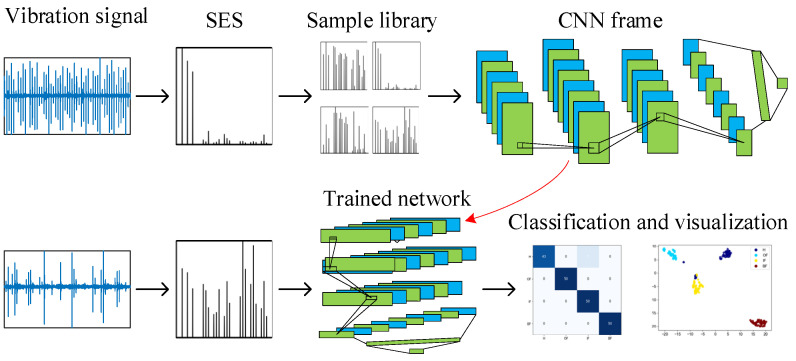
Proposed method frame.

In this paper, a deep learning method for bearing cross-domain fault diagnostics based on a reconstructed envelope spectrum is proposed, and the flow chart of the entire method is shown in Figure 3, with the main steps summarized as follows:

Step 1: Acquisition. Bearing vibration data accelerometers are used to acquire raw vibration signals from the bearings in various health states.

Step 2: Standardization processing. Standardize the original vibration signal to obtain the standard envelope spectrum of the signal.

Step 3: Standard sample library construction. A one-to-one correspondence between the standard envelope spectrum and the healthy rotational state of the bearing is used to construct a standard sample library.

Step 4: Model training. The CNN model is constructed, the standard sample library is divided into the training set, validation set and test set in the ratio of 7:2:1 to train the model, and the network model is optimally updated according to the training results to obtain the rolling bearing fault identification model.

Step 5: Fault type identification. The vibration data under different speeds and different bearing types are fed into the trained model as a test set to obtain the fault classification and visualization results.

## 4. Experimental Validation

In this section, a series of cross-domain tasks are designed, using two publicly available datasets and a dataset obtained from self-experimentation, to validate the effectiveness of the method proposed in this paper and to compare its performance with that of some popular signal preprocessing methods in terms of classification accuracy and generalization ability.

### 4.1. Experiment Setup and Data Description

In this paper, vibration signals from three devices were collected for analysis. The bearing parameters of each device are given in Table 2, and a detailed description of the data used is given in Table 3.

**Figure 4:**
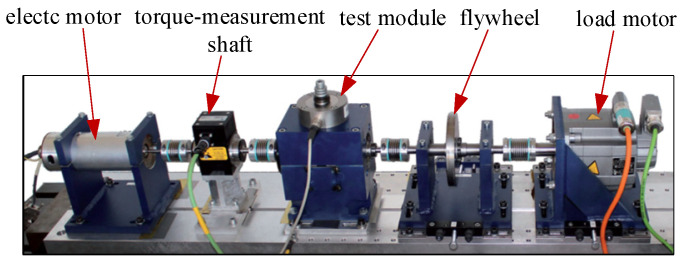
Modular test rig.

**Figure 5:**
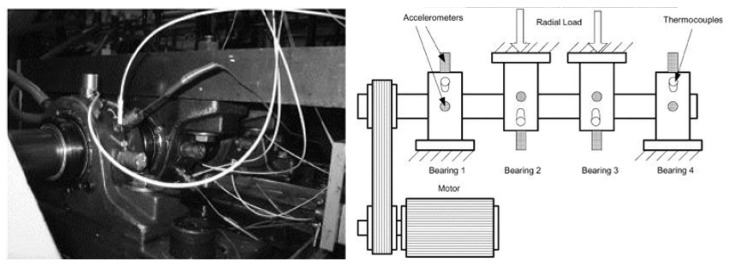
Bearing test rig.

**Figure 6:**
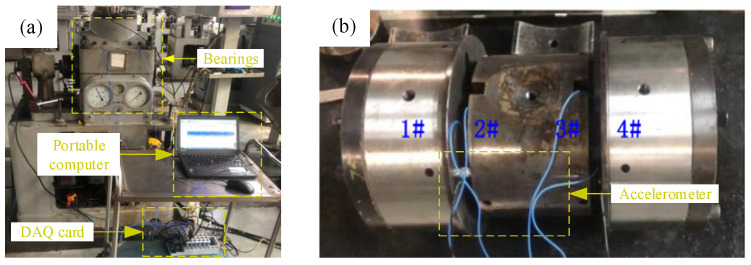
Bearing life test device: (**a**) Bearing test rig; (**b**) Bearing arrangement.

**Figure 7:**
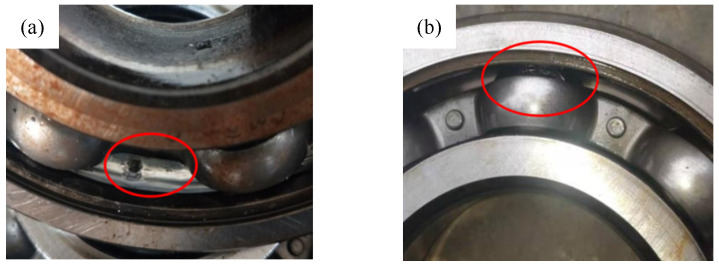
Bearing fault: (**a**) Bearing with outer race fault; (**b**) Bearing with roller fault.

A brief description of the dataset is given as follows:

(1) Data set A was obtained from the University of Paderborn, Germany [19], and the test rig included an electric motor, a torque measurement shaft, a rolling bearing test module, a flywheel, and a load motor, as shown in Figure 4. The data set includes normal bearing data and defective bearing data, in which the defective bearings are obtained by both manual machining damage and accelerated life test damage, including three fault states: an outer ring fault, an inner ring fault, and a compound fault. Each bearing is carried out under four working conditions, there are 20 data points for each working condition, each data acquisition time was 4 s, and the sampling frequency was 64 kHz. The bearing data was under a torque of 0.7 Nm, a radial load force of 1000 N, and rotational speeds of 1500 rpm and 900 rpm were selected for this validation.

(2) Data set B is from the Intelligent Maintenance System (IMS) [20]. The test rig is shown in Figure 5, with four Rexnord ZA-2115 double row bearings mounted on a shaft. The data is the full life data of the bearings and consists of three data sets, each containing the vibration data of the four bearings. The speed of the bearings was 2000 rpm, the sampling frequency was 20 kHz, the sampling interval was 10 min, the sampling time was 1 s, and a data file with 20,480 sampling points was generated. Data set 1 contains 2156 files, where bearing 3 ran until the inner ring was damaged and bearing 4 ran until the rolling element was damaged. Data set 2 contains 984 files, where bearing 1 ran until the outer ring was damaged.

(3) Data set C is the real failure data of the bearings obtained by conducting full life tests of the bearings. A total of four sets of bearings were installed on the test rig, and acceleration sensors were used to collect vibration signals. The arrangement of the test rig and measurement points is shown in Figure 6. The test speed was 3120 rpm, the sampling frequency was 12,800 Hz, the sampling interval was 1 min, the sampling time was 1.28 s, and 1 data file was generated. As shown in Figure 7a, bearing 1 ran until the rolling element was damaged, resulting in 5570 data files. As shown in Figure 7b, bearing 4 ran until the outer ring was damaged, resulting in 2473 data files.

**Figure 8:**
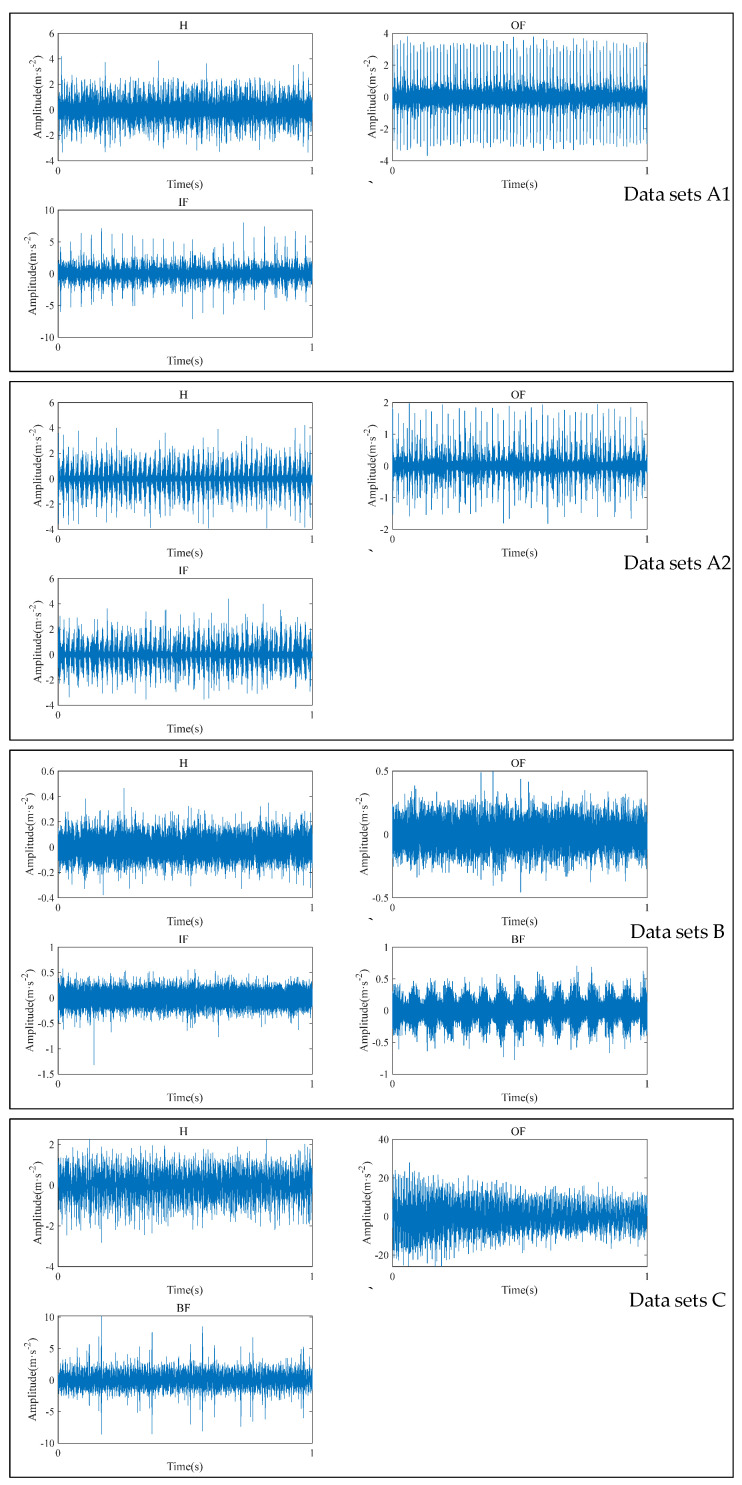
The time domain waveforms of the bearing datasets.

To ensure that the envelope spectrum has sufficient frequency resolution, the acquisition time for each of the three data sets above was assumed to be 1 s for each sample. Some of the raw time domain waveforms for the three datasets are shown in Figure 8, indicating that the same health states in the three datasets have nothing in common in the time domain.

### 4.2. Analysis of Standard Envelope Spectrum (SES)

**Figure 9:**
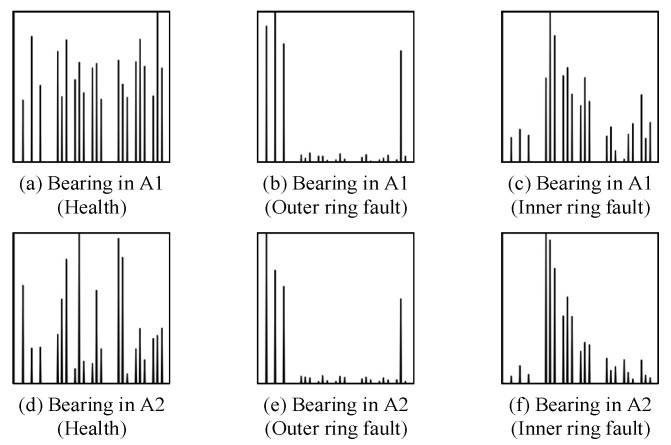
Standardized envelope spectra for different health conditions for data sets A1 in (**a**–**c**) and A2 in (**d**–**f**).

**Figure 10:**
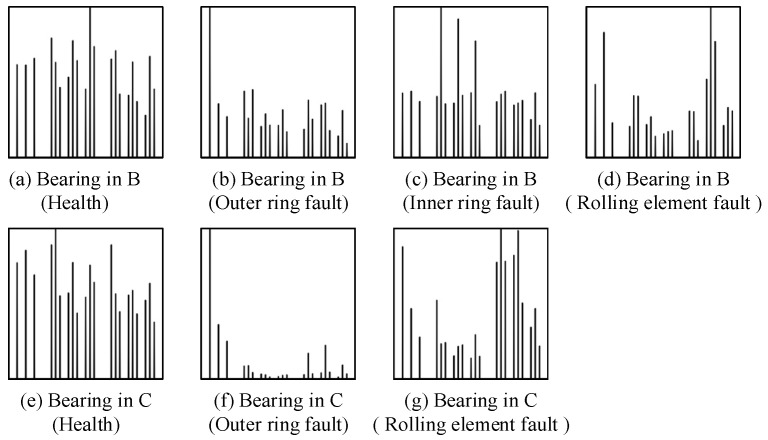
Standardized envelope spectra for different health conditions for data sets B in (**a**–**d**) and C in (**e**–**g**).

Data sets A1 and A2 are data from the same bearing at different speeds, and the standardized envelope spectra of some of their different health data are shown in Figure 9. Data sets B and C are from two different types of bearings, and their standardized envelope spectra of some different health data are shown in Figure 10. It can be clearly seen that the SES plot shows unique features for a given state of health.

The standard envelope spectrum of the same bearing at the same speed for different health states is shown in Figure 10a–d. As shown in Figure 10a, for the normal condition, the spectral lines in the three characteristic regions are prominent. This is because the normal bearing does not have cyclic shocks caused by defects, does not have prominent peaks in the envelope spectra, and there is not much difference in the magnitude of the spectral lines after normalization. As shown in Figure 10b, for the outer ring fault, the amplitude of the spectral lines in the characteristic region of the outer ring fault is prominent, which is because the amplitude at the characteristic frequency of the outer ring fault and its harmonics in the envelope spectrum are prominent. As shown in Figure 10c, for the inner-circle fault, the amplitude of the spectral lines in the characteristic region of the inner-circle fault is prominent because the amplitude at the characteristic frequency of the inner-circle fault and its harmonics and side frequencies are prominent in the envelope spectrum. As shown in Figure 10d, for the rolling body fault, the amplitude of the spectral lines in the characteristic region of the rolling body fault is prominent because the amplitude at the characteristic frequency of the rolling body fault and its harmonics and side frequencies are prominent in the envelope spectrum.

As shown in Figure 9, for data from the same bearing at different speeds, the standard envelope spectrum for the same flaw type has a high degree of similarity, and both have more prominent spectral line amplitudes in the characteristic region of the corresponding flaw type. Similarly, as shown in Figure 10, the standard envelope spectrum of the same fault type for the data from two different types of bearings also has a high degree of similarity. This indicates that the standard envelope spectrum effectively reveals the signal characteristics of rolling bearings that do not change with speed and model, but only due to changes in health.

In fact, the envelope spectrum patterns of the bearings are different, for example, some faults have prominent amplitudes for the first three harmonics of the fault characteristic frequency, some have prominent amplitudes for only one harmonic of the fault characteristic frequency, some inner ring/rolling element faults have no sidebands, and so on. At this point, it is necessary for the CNN to synthesize the spectral line conditions of the three fault characteristic regions of the standard envelope spectrum to learn and summarize the common characteristics of the corresponding health states. It is also necessary to utilize multiple bearing fault data sets as much as possible to construct a standard sample library, enrich the standard envelope spectrum form of the standard sample library, so that when it is used for new diagnostic data, the most similar standard envelope spectrum from the sample library can be easily found, and its corresponding health state is the diagnostic result.

### 4.3. Fault Diagnosis under Different Domains

In this section, five cross-domain diagnostic tasks are designed to verify the cross-domain diagnostic performance of the method proposed in this paper. Tasks 1 and 2 are to use the data of one rotational speed as the training set (source domain) and the data of another rotational speed as the test set (target domain) on the data of the same type of bearing. Tasks 3–6 are training and test sets of data from different bearing models, where Task 6 uses data from two different bearing models as the training set. For example, B → C means that data set B is used for training and data set C is used for testing. For comparison, the dataset used for testing uses 50 samples for each health state. The dataset details are shown in Table 2.

#### 4.3.1. Comparison with Time–Frequency Analysis Methods

**Figure 11:**
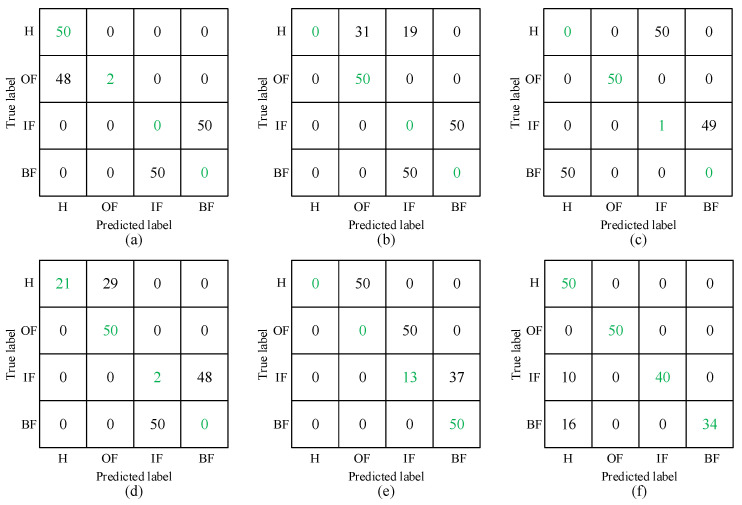
Confusion matrix of five methods in Task 6: (**a**) STFT; (**b**) WT; (**c**) VMD-HT; (**d**) MDFVI; (**e**) CSCoh; (**f**) SES.

Time–frequency analysis describes the correlation between the time and frequency domains of a signal and is an effective tool for dealing with non-stationary and transient signals [21]. The time–frequency transformed time–frequency map as a two-dimensional image is often used as input to CNN-based diagnostic models. Therefore, this paper compares the diagnostic effectiveness of STFT spectrograms, CWT spectrograms [22], VMD-HT spectrograms [23], MDFVI spectrograms [13], CSCoh spectrograms [14], and the proposed SES spectrograms as inputs. Each task is repeated 10 times, and the average diagnostic accuracy using the proposed SES-CNN method is shown in Table 3, and the results using other methods are also shown in Table 4. Task 6 is run once and its confusion matrix is shown in Figure 11, where the green font represents the number of correctly classified samples.

It can be seen that the classification accuracy of the methods used for comparison is low. This is as expected, due to the large difference between the domains caused by the different bearing speeds and bearing models, the feature extraction methods used for comparison cannot reduce this difference, and it is difficult for deep learning to learn the common features between the source and target domains without the target domain samples used for training. Compared with these methods, the method proposed in this paper shows higher classification accuracy in experiments, which indicates the effectiveness of its feature extraction, as shown in Figure 11, where most of the samples in the target domain are correctly classified.

#### 4.3.2. Feature Visualization Analysis

**Figure 12:**
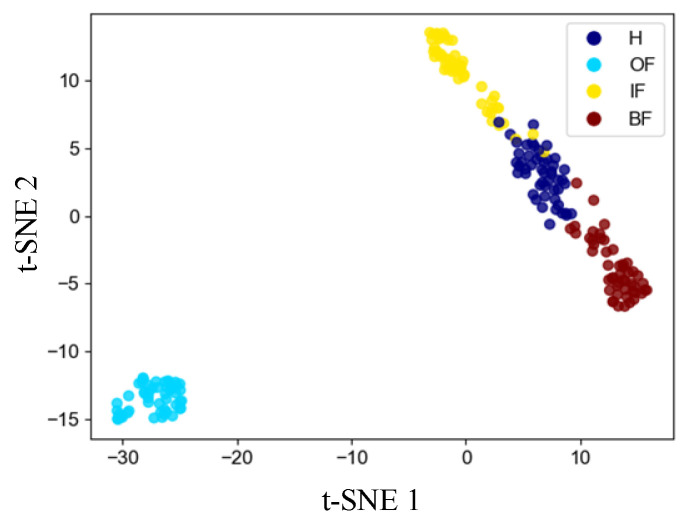
Features visualization based on t-SNE.

To further illustrate the feature learning effectiveness of the proposed method for cross-domain diagnostics, the performance of the proposed SES method on Task 6 is feature visualized using the t-distributed stochastic neighborhood embedding (t-SNE) technique [24], which maps high-level outputs from 128 to 2 dimensions in Layer 10. The results are shown in Figure 12. The four different colors indicate four different categories. It is clear from the visualization results that data points with different health states are well separated and data points with the same health state are clustered together. In addition, a small number of data points overlap between H and IF and between H and BF, suggesting that a small number of IF and BF samples are incorrectly classified as H samples, which would lead to reduced diagnostic accuracy. This observation is consistent with the results shown in Figure 11. Overall, for the untrained data of different bearing models in Task 6, the proposed method can clearly distinguish different categories. Therefore, it can be concluded that the proposed method is able to learn better discriminative features and shows strong cross-domain diagnostic capability.

## 5. Conclusions

In order to improve the cross-domain fault diagnostic capability of rolling bearings, this paper proposes a new deep learning-based fault diagnostics framework that combines SES and CNN. Cross-domain diagnostic experiments are carried out on three different sets of equipment, and the following conclusions can be drawn from the analyses of the experimental results:

(1) The constructed SES, as a pre-processing step, reveals the signal characteristics of rolling bearings that do not change with rotational speed and model number, but only due to changes in health status.

(2) The SES eliminates the differences between the source and target domains due to the different rotational speeds and models, which greatly reduces the difficulty the CNN has in learning the common features between the two different domains and improves the diagnostic performance.

(3) In the cross-domain task between six different rotational speeds and between different bearing models, the proposed method shows high classification accuracy in cross-domain diagnostics compared with some popular pre-processing methods.

## Figures and Tables

**Table 1:** Parameters of the proposed CNN architecture.

Layer	Layer Types	Kernel	Number of Filters	Filter Size	Stride	Output Size	Activation Function
1	Input					64 × 64 × 1	
2	Conv	Kernel	16	3 × 3	(1,1)	64 × 64 × 16	ReLU
3	BN					64 × 64 × 16	
4	MaxPool	Pooling size	16	2 × 2	(2,2)	32 × 32 × 16	ReLU
5	Conv	Kernel	32	3 × 3	(1,1)	32 × 32 × 32	
6	BN					32 × 32 × 32	ReLU
7	MaxPool	Pooling size	32	2 × 2	(2,2)	16 × 16× 32	
8	Flatten					8192	
9	FC					128	ReLU
10	FC					4	Softmax

**Table 2:** Data set bearing parameters.

Data Set	Type	Number of Balls (z)	Roller Diameter d (mm)	Pitch Diameter D (mm)	Contact Angle α (°)
A	6203	8	6.75	29.05	0
B	Rexnord ZA-2115	16	8.4	71.5	15.17
C	6312/C3	8	22	94	0

**Table 3:** Data set specification.

Data Set	Set	Data Number	Rotational Speed	Sample Number	Class Label
PU Bearings	A1	H: K001, K002, K003, K004, K005, K006	1500 rpm	480	0
		OF: KA01, KA09,KA04,KA16		320	1
		IF: KI01, KIO3, KI04, KI16, KI17, KI18		480	2
	A2	H:K001, K002, K003, K004, K005, K006	900 rpm	480	0
		OF:KA01, KA09, KA04, KA16		320	1
		IF:KI01, KIO3, KI04, KI16, KI17, KI18		480	2
IMS Bearings	B	H: Data set 2, Bearing 1, Filess 1–200	2000 rpm	200	0
		OF: Data set 2, Bearing 1, Files 513–712		200	1
		IF: Data set 1, Bearing 3, Files 2056–2155		100	2
		BF: Data set 1, Bearing 4, Files 1757–1956		200	3
Experimental bearings	C	H: Bearing 1, Files 1–300	3120 rpm	300	0
		OF: Bearing 4, Files 501–800		300	1
		BF: Bearing 1, Files 3001–3300		300	3

**Table 4:** Comparison results of different input features under different cross-domain tasks.

No.	Source → Target	STFT	CWT	VMD-HT	MDFVI	CSCoh	SES
1	A1 → A2	67.33%	67.33%	34.67%	74.67%	65.33%	98.67%
2	A2 → A1	67.33%	66.67%	33.33%	88.00%	70.00%	92.00%
3	B → A1	34.0%	34.67%	33.33%	66.67%	34.00%	94.00%
4	B → A2	33.33%	33.33%	34.00%	54.67%	33.33%	95.33%
5	B → C	34.00%	33.33%	34.67%	49.33%	33.33%	90.00%
6	A + C → B	26.00%	25.00%	25.50%	36.50%	31.50%	87.00%

## Data Availability

Data set A was obtained from the University of Paderborn, Germany, original data download link: https://blog.csdn.net/ynn4818172/article/details/122755894 (accessed on 10 April 2024). Data set B is from the Intelligent Maintenance System (IMS), original data download link: https://ti.arc.nasa.gov/tech/dash/groups/pcoe/prognostic-data-repository/#bearing (accessed on 10 April 2024).
